# Whole-cell vaccine candidates induce a protective response against virulent *Acinetobacter baumannii*

**DOI:** 10.3389/fimmu.2022.941010

**Published:** 2022-09-27

**Authors:** Stephen J. Dollery, Daniel V. Zurawski, Ruth V. Bushnell, John K. Tobin, Taralyn J. Wiggins, David A. MacLeod, Naomi J. P. E. R. Tasker, Yonas A. Alamneh, Rania Abu-Taleb, Christine M. Czintos, Wanwen Su, Mariel G. Escatte, Heather N. Meeks, Michael J. Daly, Gregory J. Tobin

**Affiliations:** ^1^ Biological Mimetics, Inc., Frederick, MD, United States; ^2^ Wound Infections Department, Bacterial Diseases Branch, Center for Infectious Disease Research, Walter Reed Army Institute of Research, Silver Spring, MD, United States; ^3^ Defense Threat Reduction Agency, Fort Belvoir, VA, United States; ^4^ Department of Pathology, Uniformed Services University of the Health Sciences, Bethesda, MD, United States

**Keywords:** pulmonary, protection, *A. baumannii*, humoral, vaccine, whole-cell, UVC, MDP

## Abstract

*Acinetobacter baumannii* causes multi-system diseases in both nosocomial settings and a pre-disposed general population. The bacterium is not only desiccation-resistant but also notoriously resistant to multiple antibiotics and drugs of last resort including carbapenem, colistin, and sulbactam. The World Health Organization has categorized carbapenem-resistant *A. baumannii* at the top of its critical pathogen list in a bid to direct urgent countermeasure development. Several early-stage vaccines have shown a range of efficacies in healthy mice, but no vaccine candidates have advanced into clinical trials. Herein, we report our findings that both an ionizing γ-radiation-inactivated and a non-ionizing ultraviolet C-inactivated whole-cell vaccine candidate protects neutropenic mice from pulmonary challenge with virulent AB5075, a particularly pathogenic isolate. In addition, we demonstrate that a humoral response is sufficient for this protection *via* the passive immunization of neutropenic mice.

## Introduction

*Acinetobacter baumannii* are Gram-negative bacteria and a causative agent of numerous life-threatening illnesses such as pneumonia, sepsis, and urinary tract infections. Carbapenem-resistant *A. baumannii* (CRAB) is of particular concern and CRAB continues to top the lists of “critical” pathogens for which new countermeasures are urgently needed as identified by numerous expert groups (1, 2). Treatment of CRAB infections is dependent upon a last line of drugs such as colistin-rifampin or carbapenem-sulbactam which can reduce mortality (3). The bacteria are evolving to be increasingly resistant to multiple antibiotics with few countermeasures in the pipeline (4–6).

*A. baumannii* infections occur in both healthcare and community settings with pneumonia being the most commonly reported condition in each. Infections frequently present in those with underlying health conditions such as diabetes and chronic obstructive pulmonary disease, but also occur following trauma, burns, and antibiotic use, or during any prolonged hospital stay (6, 7). At the end of the twentieth century, *A. baumannii* emerged as a nosocomial agent of concern with prevalence in intensive care units and on surgical implants. *A. baumannii* has been particularly troublesome for otherwise healthy, wounded military personnel with well-documented outbreaks associated with field hospitals of the U.S., U.K., and Canadian Forces (8). Although the incidence of nosocomial cases has declined due to improved disinfectant countermeasures, the CDC’s published pre-COVID-19 numbers state there were approximately 8,500 cases in hospitalized patients with over 700 deaths in the hospital setting in the U.S.A (2), and globally there are an estimated 1,000,000 cases per year (50% CRAB) (9, 10). More recently, *A. baumannii* was associated with secondary infection following SARS-CoV-2 infection as a ventilator-associated pathogen. The extent and contribution to mortality in this context are as yet not reported (11–13). To date, there has not been a single trial of a vaccine to protect against *A. baumannii* infections in humans.

There are numerous promising *Acinetobacter* vaccine candidates comprised of whole-cell, live attenuated, outer membrane vesicle (OMV), outer membrane complex (OMC), DNA, and recombinant subunit vaccine approaches (including Ata, BamA, Bap, BauA, BfnH, Blp1, CsuA/B, FilF, FimA, NucAb, Oma87, Omp22, OmpA, OmpK, OmpW, SmpA, Pal, PLD, Trx and ZnuD), many of which convey certain beneficial features which are nicely compared in recent reviews (14–18). Of these, whole-cell, OMV, and OMC vaccine candidates have thus far provided the best requisite heterologous protection in animal models of infection. In each of the whole-cell and “multi-component” vaccines reported, bacteria for immunogen generation was cultivated using a single method. In addition, whole-cell vaccines have been produced using traditional chemical fixation (19–21).

A major contributing factor to the lack of efficacy of chemically fixed vaccines is the destruction of immunogenic epitopes during the inactivation process (22, 23). However, with the discovery of a manganous-decapeptide-phosphate (MDP) complex that specifically protects proteins, but not nucleic acids, from reactive oxygen species (ROS), we have uncoupled genome destruction from damage to epitopes during irradiation (24–27). The methodology was derived through the discovery of ROS-scavenging mechanisms by which the radioresistant bacterium *Deinococcus radiodurans* evolved to survive desiccation (28–30). Briefly, *D. radiodurans* hyperaccumulates manganous-peptide-phosphate complexes named *Mn antioxidants* which specifically protect proteins from oxidation to preserve the functionality of enzymes needed to repair DNA damaged by radiation (28, 30, 31). Daly et al. reconstituted the protective complex by combining MnCl_2_ with a rationally-designed decapeptide (DEHGTAVMLK) in a potassium phosphate buffer (32). More recently, our group has broadened the applicability of the MDP-irradiation platform with the identification of lead vaccine candidates against poliovirus (25), *A. baumannii* (24), and MRSA (33). Our recent findings with *A. baumannii* showed that a gamma radiation-inactivated whole-cell vaccine prepared in the presence of MDP afforded 100% protection against pulmonary challenge in healthy mice, and, as anticipated, that some cultivation methods generated better immunogens than others when compared in neutropenic mice (24). Several candidates remained 80-90% protective in a neutropenic pneumonia model when challenged with a notably virulent, and extensively drug-resistant (XDR) isolate (AB5075), which to our knowledge is unprecedented (24).

In this report, we present the results of studies on radiation-inactivated whole-cell vaccine candidates for *A. baumannii*. The most salient features of our results include the substitution of ultraviolet C (UVC)-irradiation for gamma-irradiation as an easier, more accessible inactivation method, and observations that *A. baumannii* immunogens inactivated by UVC with or without the MDP complex were equivalent using a high dose, two boost vaccination regimen. Finally, we demonstrate that the humoral response is sufficient for the protection of neutropenic mice using passive vaccination with immune sera.

## Materials and methods

### Bacterial strains and cultures

The XDR AB5075 strain was used in these experiments. Planktonic #1 (P1) and biofilm #1 (B1) forms were identified in previous studies as stimulating protection in mouse challenge studies (24). Briefly, B1 cultures were propagated for 3 days at 37 °C on polystyrene substrates submerged in stationary T182 tissue culture flasks using complete M9 media supplemented with 2 mM MgSO_4_, 0.4% glucose, 0.1 mM CaCl_2_, and 0.1% casamino acids. At 24 and 48 hr after inoculation, spent culture broth was removed, the flasks were rinsed with phosphate-buffered saline (PBS) to remove non-adherent cells, and 50 mL of fresh complete M9 media was introduced into the flasks. After 72 hr, non-adherent cells were again removed by rinsing with PBS. Adherent cells were harvested into 10 mL volumes of PBS by manual removal with plastic scrapers. The cells were pelleted by centrifugation (2,000 x *g* for 20 min) and resuspended in small volumes of PBS. P1 cultures were propagated for 20 hours at 37°C in shaking flasks using Tryptic Soy Broth (TSB) after which the cells were pelleted by centrifugation, washed three times with PBS, and resuspended in small volumes of PBS. The washed bacterial preparations were stored at 4°C for up to 14 weeks without detectable degradation (24).

### Inactivation of bacterial replication

The synthetic peptide (DP1) H-Asp-Glu-His-Gly-Thr-Ala-Val-Met-Leu-Lys-OH (27) was custom synthesized and refined to 95% purity by GenScript, Inc., Piscataway, NJ. The B1 and P1 cells were compounded as 0.2 mL samples at densities of 10^9^ to 10^11^ CFU/mL and concentrations of 3 mM DP1, 25 mM potassium phosphate, pH 7.4, and 1 mM MnCl_2_. All gamma irradiations were performed on wet ice (0 °C) in 0.5 mL o-ring polypropylene tubes on ice using a ^60^Co source emitting between 10 and 15 kGy/h. UVC irradiations were performed in 0.2 mL thin-wall polypropylene tubes at room temperature using a UVC source emitting between 4 and 4.5 mW/cm^2^ (Analytik Jana US, UVG-54). Similar samples were prepared for irradiations performed without MDP except that both DP1 and MnCl_2_ were omitted. After end-point irradiation, at least 10^10^ colony forming units (CFU)-equivalent samples of bacteria were cultured overnight on LB agar plates to assess the presence of residual replicative activity. Immunogens were stored at 4 °C until immunization. Graphing and calculations of standard error of the mean were performed in Excel version 2206.

### Analysis of protein profiles

Bacterial cultures were analyzed by Coomassie-stained gels and western blots. Approximately 5 x 10^8^ CFU of bacteria were mixed with equal volumes of 2x Laemmli SDS-PAGE sample buffer (BioRad, Hercules, CA), denatured by heating 15 min at 100 °C, and centrifuged at 16,000 x g for 5 min to clarify the crude lysates. Samples were electrophoresed in denaturing polyacrylamide gels (4-20% acrylamide). For total protein analysis, the gels were stained with Bio-Safe Coomassie G-250 stain (Bio-Rad) and digital images taken with a cell phone camera. For western blots, proteins in the gels were transferred electrically to nitrocellulose using a Bio-Rad Trans-Blot Turbo Blotting System. The membranes were pre-treated with a solution of 10% non-fat dried milk in PBS to block non-specific antibody binding and then probed with pooled sera from mice immunized with AB5075 (section 2.5). Following a wash step with PBS-T (PBS with 0.1% Tween-20), the blots were reacted with goat anti-mouse antibody conjugated with horseradish peroxidase. The blots were washed again and developed in chemiluminescent substrate (Clarity Western ECL Reagent, BioRad, CA). Antibody-reactive proteins were visualized by exposure to x-ray film.

### Analysis of oxidative damage

B1 and P1 cultures were inactivated with and without the MDP complex to assess its role in protecting proteins from oxidative damage during irradiation. Carbonyl groups in the bacterial proteins were derivatized to 2,4-dinitrophenylhydrazone by reaction with 2,4-dinitrophenylhydrazine (DNPH) for 15 min in 3% (w/v) SDS using the OxyBlot™ Protein Oxidation Detection kit (Chemicon International). After separation by denaturing polyacrylamide gels and electroblot to nitrocellulose, the membranes were probed with anti-DNP antibody, washed, and reacted with HRP-conjugated goat anti-rabbit IgG. Reactive proteins were visualized by use of chemiluminescent substrate (SuperSignal, Pierce Biotechnology, Rockford, IL) and imaged by exposure to x-ray film.

### Production of immune sera for passive immunity

Two groups of ten BALB/c mice were immunized with 1 x 10^8^ B1 and P1 samples that had been complexed with MDP and inactivated with gamma irradiation. The mice received intramuscular immunizations on Days 1 and 21 and were then euthanized humanely for the production of immune sera.

### Mouse model of pulmonary challenge

All mouse studies were conducted in accordance with the Guide for the Care and Use of Laboratory Animals (National Research Council, 2011), and procedures were approved by the Institutional Animal Care and Use Committee at the Walter Reed Army Institute of Research (WRAIR) (protocol# 20-BRD-29). The murine pulmonary model was used as previously described (34).

BALB/c mice were immunized into one quadriceps with an equal number of inactivated B1 and P1 bacterial cells correlating to 1.0 x 10^7^ CFU each in 100 μL volumes without adjuvant. Four and six weeks later, similar booster immunizations were performed. Two weeks after the final boost, the animals were injected with cyclophosphamide 4 days and 1 day before the intranasal challenge with 5.0 x 10^6^ CFU of AB5075 in sterile saline (LD80) (34). In both sets of experiments, mice were observed for one week, and each experiment with 5 mice/group was repeated at least once (n = 10 mice/condition). One set of mice was injected with serum one day before challenge *via* the intraperitoneal route (i.p.) at the same time as the cyclophosphamide injection. Mice were weighed each day and monitored for morbidity and mortality with clinical scoring each day, twice or three times a day for seven days. Mice were humanely euthanized with CO_2_ inhalation at the experiment’s end or when clinical scoring and weight loss (>25%) suggested they were succumbing to bacterial infection. The Kaplan–Meier estimate was used to measure survival over time. The significance of differences between groups was analyzed using the log-rank (Mantle–Cox) test using Prism version 8.0.0. (San Diego, CA, USA). Holm-Bonferroni corrections for multiple comparisons of Mantle-Cox p-values were calculated in Excel version 2206.

### enzyme linked immunosorbent assay (ELISA) using anti-*A. baumannii* sera

1x10^7^ CFU equivalents of MPD-inactivated B1 or 3 x 10^7^ CFU equivalents of MDP-inactivated P1 *A. baumannii* diluted in waster were used to coat Nunc Maxi Sorp high-binding ELISA plates overnight. The plates were washed in ELISA wash buffer (PBS with 0.1% Triton X-100) and blocked for 2h in a solution of wash buffer with 5% non-fat dried milk. The plates were washed again and incubated with serial dilutions of sera from mice immunized with MDP-inactivated P1 or B1 or a 50:50 mixture of both sera. Following a 1h incubation at room temperature, the plates were washed and probed with goat-anti-mouse-HRP (Kirkegaard & Perry Laboratories, Inc.) at a 1:1k dilution for 1h at room temperature. Plates were washed and incubated with 3,3’,5,5’-tetramethylbenzidine substrate. The reaction was stopped by the addition of 1N HCl. Plates were read at 450 nm using a SpectraMax 340 and recorded using SoftPromax software 3.1.2.

## Results

### Inactivation kinetics

**Figure 1:**
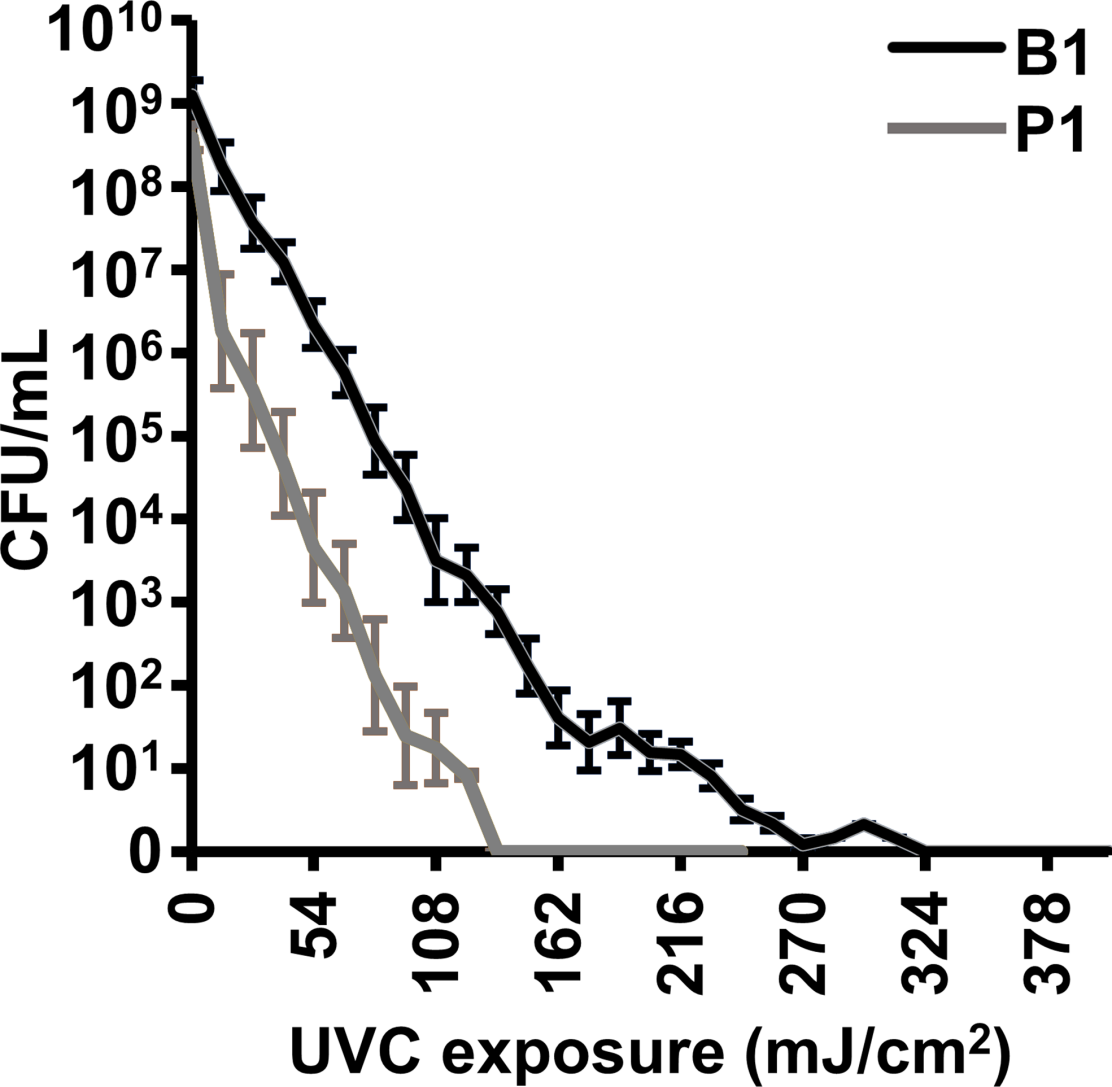
UVC inactivation of *A. baumannii* vaccine candidates B1 and P1. 0.1 mL volumes of bacterial cultures at approximately 1 x 10^9^ CFU/mL were compounded with the MDP complex and exposed to UVC irradiation (4.5 mW/cm^2^) for increasing times. Energy is shown in millijoules (mJ/cm^2^). The treated samples were serially diluted and plated to determine the concentration of live, colony-forming, bacteria. The experiment was done in triplicate and error bars show the standard error of the mean.

Inactivation parameters for AB5075 cultures using gamma-irradiation have been previously described and were followed for immunogen generation. In brief, it was determined that no residual replicative activity remained in cultures after 8 kGy gamma irradiation (24). As a safety feature, all gamma-irradiated bacterial samples used for immunization were exposed to 10 kGy. The B1 and P1 bacteria were compounded with MDP and exposed to UVC irradiation for increasing lengths of time to determine the minimum exposure for reliable inactivation of replicative capability. As can be seen in **Figure 1**, the planktonic P1 sample was more sensitive than the biofilm B1 sample to UVC inactivation. To provide a safety margin, P1 samples were inactivated using 45 second (~200 mJoules) exposures and B1 samples 90 second (~400 mJoules) exposures (approximately 50% over inactivation dose). To ensure that mice were not injected with live bacteria, samples containing at least 1 x 10^9^ CFU of UVC-exposed bacteria were spread on LB-agar plates lacking antibiotics. After overnight incubation at 37 °C, the plates were inspected visually to confirm that no colony-forming activity was present. No colonies were visible on plates incubated for longer periods).

### Global analysis of immunogen protein profiles post-UVC inactivation

**Figure 2:**
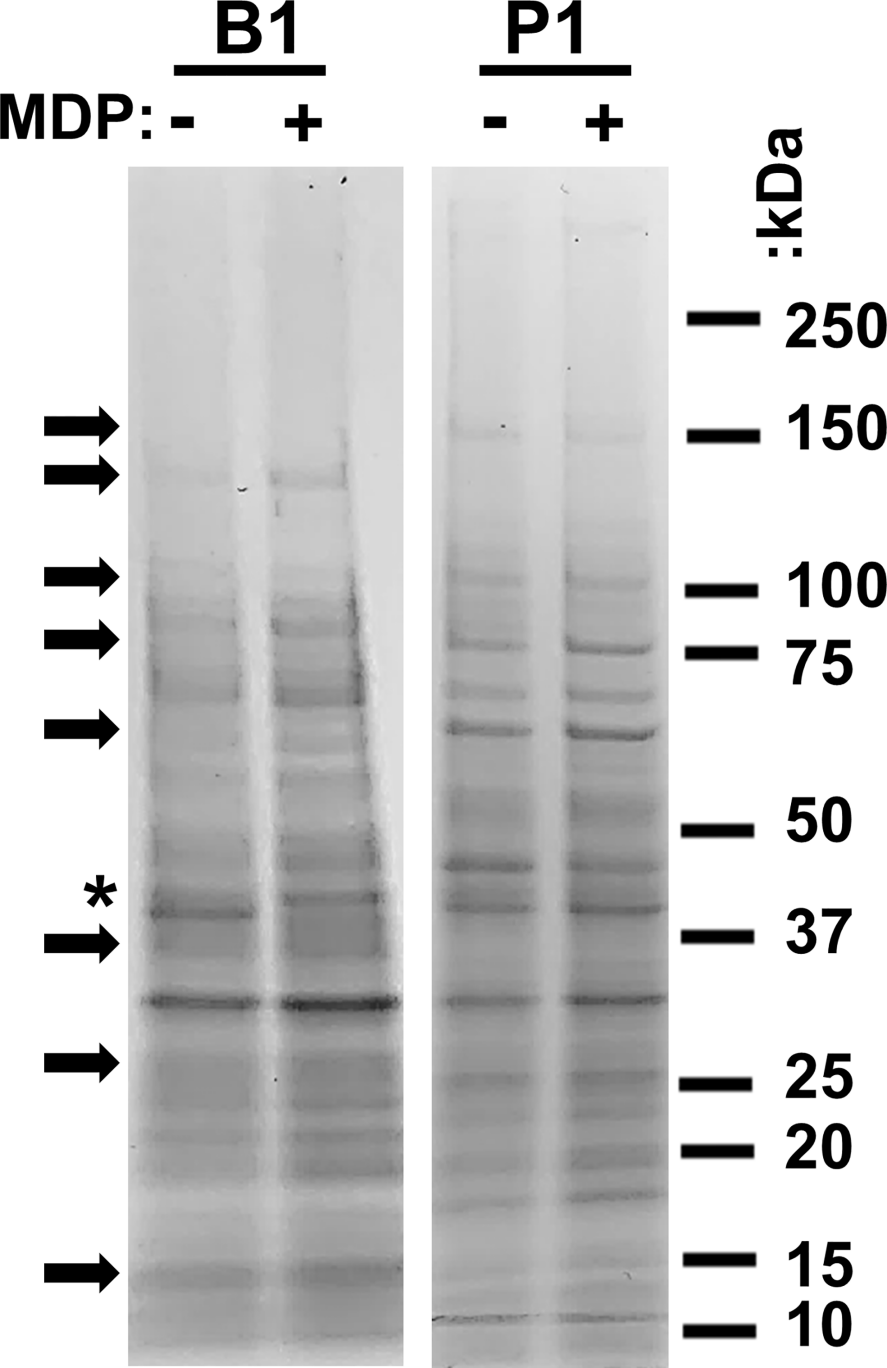
Analysis of immunogen protein profiles. B1 and P1 cultures of AB5075 were inactivated with UVC with (+) and without (–) the protective MDP complex. 1 x 10^8^ bacterial cells were denatured in SDS-PAGE sample buffer and electrophoresed in a 4-20% polyacrylamide gel. Samples were then stained with Coomassie Brilliant Blue to visualize the total protein profiles of the four samples. Migration of molecular weight markers is shown to the right of the figure. Arrows indicate prominent differences between the B1 and P1 preparations. An asterisk highlights the one prominent exception at 40 kDa in samples prepared without MDP.

Bacterial cultures adapt to their growth environment and express different protein profiles accordingly. We have previously observed that there are numerous differences between the protein profiles of P1 and B1 samples and that gamma irradiation in the presence of MDP is not detrimental to the majority of epitopes (24). We sought to establish if the presence of MDP during UVC irradiation altered the global protein profile of the immunogens. B1 and P1 cultures were washed in PBS and exposed to UVC irradiation with and without the MDP complex. Samples were then denatured and analyzed by SDS-PAGE (**Figure 2**). As expected, many proteins appear upregulated and down-regulated between the P1 and B1 preparations (several are highlighted with black arrows). The overall composition of the preparations appears equivalent for samples made in the presence or absence of MDP with minor differences in comparative concentration seen for some individual bands. One exception is a protein with an approximate mass of 40 kDa seen in the B1samples prepared without MDP.

### MDP protects *A. baumannii* from oxidative damage during UVC exposure

**Figure 3:**
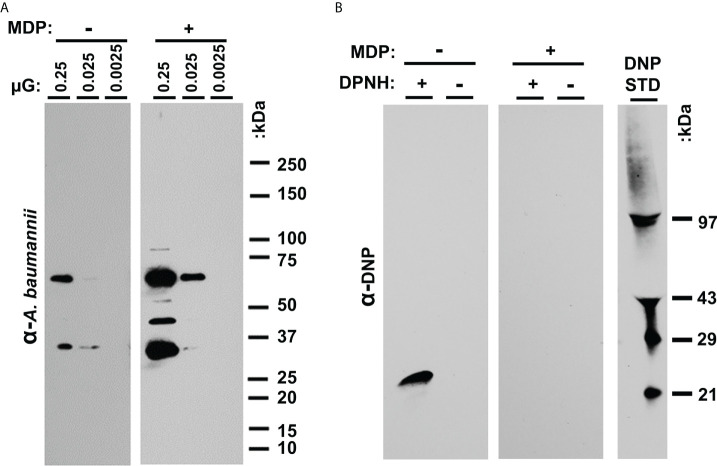
** (A)** Analysis of UVC-irradiated immunogens with and without MDP. Western analysis of epitopes. P1 samples irradiated in the presence and absence of MDP were loaded at the indicated concentration (approx. 2.5 x 10^7^ CFU in lane 1) in replicate. **(B)** Carbonylation analysis. B1 and P1 samples were UVC inactivated with and without the inclusion of the MDP Mn-antioxidant complex. After reacting the proteins with DNPH, the samples were denatured, electrophoresed, and transferred to nitrocellulose. The membranes were probed with anti-DNP antibody and detected with HRP-conjugated secondary antibody.

To further evaluate immunogens prepared with UVC in the presence and absence of MDP, epitope recognition with immune sera and analysis of protein oxidation was performed. Sero-reactive proteins from P1 samples were examined by western blot using pooled sera from mice immunized with B1 and P1 cells (**Figure 3A**). In the highest concentration lane, numerous bands appear to be protected by MDP. Densitometry (ImageJ) reveals over 600% more signal is detected in preparations irradiated in the presence of MDP. Daly *et al.*, 2007 correlated protection against protein oxidation as a primary determinant of radio-resistance across various bacterial species. The extent of carbonylation, a measure of oxidative damage to amino acid side groups, can be detected by derivatizing carbonyl groups in amino acid side chains to 2,4-dinitrolphenlylhydrazone (DNP) by reaction with 2-4-dinitrolphenyldyrazine (DNPH). DNP is then detectable with DNP specific antibody. To determine whether the presence or absence of MDP during UVC exposure altered the oxidation state of whole-cell *A. baumannii* immunogens, P1 preparations were UVC-inactivated with or without MDP and carbonyls derivatized to DNP. A western blot for DNP is shown in **Figure 3B** that shows oxidation was only detectable in the derivatized (+DNPH) sample in the absence of MDP.

### Immunogens and mice

**Figure 4:**
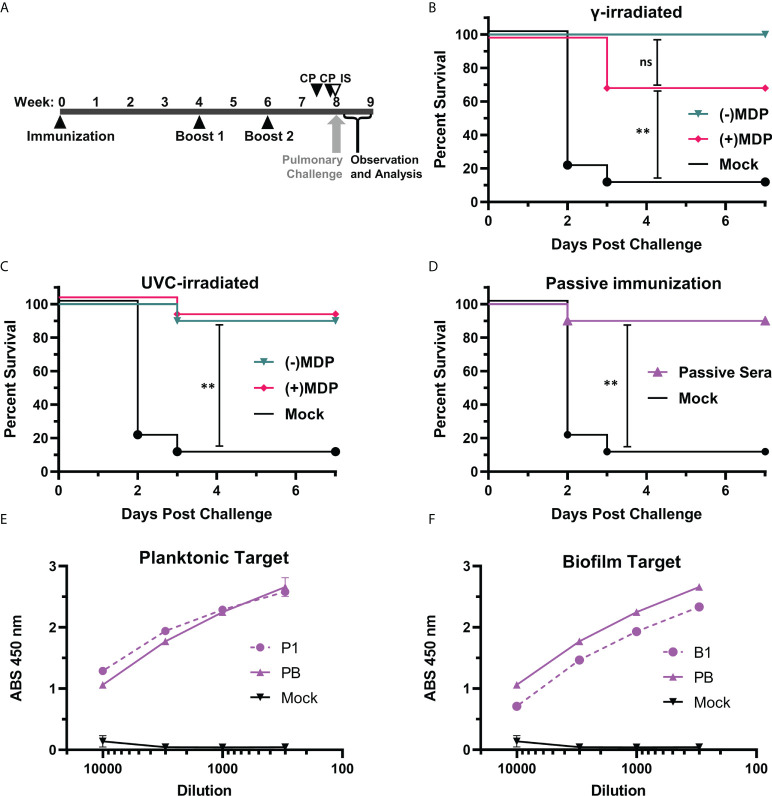
Pulmonary challenge in neutropenic mice. **(A)** Vaccination schedule. BALB/c mice, two independent groups of five (10 total/per group) were vaccinated and boosted at weeks 4 and 6 post vaccination. Cyclophosphamide (CP) was given at days -4 and -1 pre-challenge. In one group, immune serum (IS) was given 1 day before pre-challenge *via* i.p. injection with cyclophosphamide treatment. Mice were challenged at 8 weeks *via* droplet exposure and observed for one week**. (B)** Gamma-Irradiated P1B1. Survival analysis of challenged mice vaccinated with immunogens prepared *via* gamma irradiation in the presence or absence of MDP. **(C)** UVC-Irradiated-P1B1. Survival analysis of challenged mice vaccinated with immunogens prepared *via* UVC-irradiation in the presence or absence of MDP **(D)** Passive immunization. Survival analysis of mice given sera from P1 and B1 vaccinated mice. Log-rank (Mantle–Cox) test p values were calculated and include the Holm-Bonferroni correction for multiple comparisons. Values are indicated by ns, not significant (p ≥ 0.05), and **p ≥ 0.01). **(E)** Sera reactivity against *A. baumannii*. ELISA data reactivity of sera raised against P1 and a pool of P1B1 using MDP inactivated P1 as a target. **(F)** Sera reactivity against *A. baumannii*. ELISA data reactivity of sera raised against B1 and a pool of P1B1 using MDP inactivated B1 as a target.

In previous studies, we demonstrated that the gamma-irradiated MDP + P1B1 immunogen protected over >80% of mice challenged in a stringent neutropenic pulmonary model (22, 29, 34). In this study, we investigated the use of UVC irradiation as a more accessible alternative and investigated the mechanism of protection. Two separate groups of five BALB/c mice (10 total) were immunized with 1.0 x 10^7^ CFU inactivated bacterial cells and then boosted twice as per **Figure 4A**. An additional group was injected i.p. with 0.1 mL of mouse immune sera raised against the immunogens to assess passive immunity.. The mice were examined twice a day for signs of disease and pulmonary distress. Moribund mice were humanely euthanized. Observations on the number of survivors are plotted in survival graphs (**Figures 4B–D**). In **Figure 4B**, neutropenic mice that had been immunized with the gamma-irradiated P1B1 immunogen prepared in the presence or absence of MDP were protected up to 100% when challenged with AB5075. No significant difference was seen between the mice immunized with preparations irradiated in the presence or absence of MDP. In **Figure 4C** mice were immunized with immunogens inactivated *via* UVC exposure in the presence or absence of MDP. Nine out of ten mice in each group survived the challenge and no significant difference was observed between plus or minus MDP. No significant difference was observed between P1B1 immunized groups in **Figure 4B, C**. To begin to address the mechanisms of protection induced by the P1B1 immunogens, sera against P1 and B1 were raised in mice for use in passive immunizations. Sera were combined and injected into mice 1 hr prior to pulmonary challenge. **Figure 4D** shows that in a group of mice passively immunized, 90% of the mice survived pulmonary challenge with AB5075. For each of these figures, the same mock vaccinated group is shown. **Figures 4E**, **F** show ELISA data for sera raised against P1, B1, or a 50:50 mixture of P1 and B1 against MDP inactivated B1 or P1 bacterial cells. Detection at dilutions al low as 1:10,000 indicate the presence of abundant anti-*A. baumannii* antibody induced by these preparations.

## Discussion

The data herein strengthens and extends our previous observations in which we showed protection using gamma-inactivated whole-cell preparations of the *A. baumannii* bacteria (24). The data shown in **Figure 4** are especially compelling for three reasons: we first see that UVC-irradiation can produce a highly effective inactivated vaccine candidates without the addition of MDP; second, we see that UVC-inactivated vaccines are approximately equivalent (statistically inseparable) to gamma-irradiated immunogens when compared directly; third, we demonstrate that antibodies alone, in the form of passive immunization with immune serum, are sufficient to suppress a lethal pulmonary challenge with AB5075 to levels equivalent to our lead vaccine candidate. Because the i.p. injection provides a systemic route to the lungs as we have shown with small molecules (35) and monoclonal antibodies (our unpublished data) in this model, we interpret this finding to indicate that immunoglobulins at the site of challenge are effective in reducing bacterial infection and replication. We plan to follow up with post-infection administration of passive immunity as a potential treatment option. It will be interesting to compare the histopathology of vaccinated and passively immune mice.

Several *Acinetobacter* vaccine candidates have been previously reported. However, none have yet entered into clinical trials. Many subunit vaccine candidates have been shown to offer protection against challenges with heterologous strains in healthy mice (14–16). There are, however, many known challenges with developing subunit vaccines for bacteria such as the functional redundancy of many virulence factors, differentially expressed proteins, heterogenic stages of biofilm development, and quandaries over finding conserved targets amongst strains (17, 36–39). Multi-immunogen vaccines may prove more efficacious, but for *Acinetobacter*, these are in earlier stages of development (17); and for other pathogens, this has not always been successful (40). In addition to subunit vaccines, several other whole-cell and bacteria-derived vaccines have been shown to offer heterologous protection in healthy mice (19–21, 41–44); however, the correlates of protection are still not well defined. The immunogen preparation method may offer several advantages over formalin fixed immunogens as formalin damages proteins and nucleotides, and specifically proteins as an alkylating agent by reaction with carboxyl, sulfhydryl, and hydroxyl groups (45). Therefore, without intact protein and protected epitopes, formalin-killed bacteria could elicit a weaker humoral, antibody response. The combination of two different growth states likely also provides more of an immunogenic response because more proteins and carbohydrates become available as antigens. To our knowledge, the data presented in **Figure 4** are the first to show a humoral response of sufficient strength and/or quality that protection can be passively transferred *via* sera to neutropenic mice. The high antibody levels detected in **Figure 4** may hint at future correlates of protection.

It is interesting that both UVC- and gamma-inactivated vaccine preparations can protect mice given that the molecular mechanisms of cell inactivation by UVC (200-280 nm) and gamma radiation are distinctly different. Under aqueous conditions, damage to macromolecules (DNA, proteins, lipids) by ionizing radiation (x- and γ-rays) is caused mainly by the indirect effects of ROS produced by water radiolysis (30). In contrast, UVC does not generate ROS in water, but causes direct damage to respiratory proteins and induces oxidative stress in metabolically-active cells, as well as causing direct DNA damage. This mechanistic difference in ROS production during irradiation explains why the Mn antioxidant MDP is required in the preparation of some irradiated vaccines, but not others. For example, the small genomes of purified viruses demand very high ionizing radiation doses to ensure inactivation (30-60 kGy); without MDP at such extreme doses, the structural integrity of viruses is lost and proteins are irreversibly oxidized, which renders the preparations nonprotective (24, 26). Of course, bacteria have much larger genomes than viruses and are typically inactivated and will lyse at much lower irradiation doses, both ionizing and UVC forms (46). The characteristic that governs whether or not a bacterium loses structural integrity at sterilizing doses of radiation is Mn antioxidant content, as exemplified by extremely desiccation-resistant bacteria that hyperaccumulate Mn antioxidants (e.g. *Deinococcus*, *Acinetobacter*, and *Bacillus* spp.) (27, 31, 47, 48). Nonetheless, most pathogenic bacteria are desiccation-sensitive and do not accumulate radioprotective Mn antioxidants, rendering them susceptible to radiation-induced protein oxidation and cell lysis (e.g. *Escherichia*, *Pseudomonas*, and *Chlamydia* spp.). Consistent with this assessment, we show that highly protective inactivated whole-cell vaccines can be made from the extremely desiccation-resistant *A. baumannii* using UVC alone, and without the need for MDP during irradiation (28, 31). However, if the immunization dose were lowered, perhaps MDP would be required to attain the same protection observed here. We postulate that the immunization and boosts have a high enough amount of cells (1.0 x 10^7^ CFU), that enough antigens are present for a stimulating immune response even if some have degraded without the presence of MDP.

The enhanced vaccine-induced protection observed could also be tied to our previous candidate development method, where the combination of two distinct protein profiles were used to cover the many different *in vivo* growth states of the bacteria. As we had previously seen in animal models, the presence of MDP, despite some demonstrated protection of epitopes (**Figure 3**), did not alter the observed efficacy of the vaccine candidates. While it is possible that reactive oxygen species disproportionately damage non-protective epitopes, it is more likely that any benefits of MDP might be more discernable when mice are vaccinated with reduced doses of immunogen or in analyses of the broadness of protection. In both studies, the quantity and condition of epitopes within the immunogen might be critical, and the ability to use reduced doses of a whole-cell vaccine is an attractive prospect for this platform. It will be important to further characterize protective antibody responses, identify correlates of protection, and evaluate this with reduced doses in further studies. Of value for the global fight against MDR-bacteria, UVC irradiation is orders of magnitude more accessible in terms of equipment cost, availability, ease of use, and safety. We view this result as a major step forward in the advancement of an irradiation-inactivated vaccine candidate.

## Conclusion

Our findings indicate that a strong humoral response is sufficient to protect against pulmonary infection in the absence of neutrophils and other circulating monocytes. We believe the data presented clearly demonstrate the potency of the whole-cell P1B1 vaccine candidate and that further development is needed. Inactivated whole-cell bacterial vaccines present large numbers of immunogens and may represent effective platforms for protection against bacteria that exist as swarms of different antigenic variants. By screening and combining two unique whole-cell vaccine candidates, the P1B1 formulation should target multiple, stage-specific, expression profiles, which include many pan-*Acinetobacter* targets. In the wake of emerging XDR and pandrug-resistant strains, a simple vaccine approach such as this could be a solution even if further antibiotics are developed. A multi-pronged treatment/prevention approach including a vaccine will be especially important for regions of high prevalence, or future areas where resistant strains further emerge.

## Data availability statement

The original contributions presented in the study are included in the article/supplementary material. Further inquiries can be directed to the corresponding author.

## Ethics statement

The animal study was reviewed and approved by Institutional Animal Care and Use Committee at the Walter Reed Army Institute of Research (WRAIR) (protocol# 20-BRD-29).

## Author contributions

Conceptualization, DZ, HM, MD and GT; data curation, SD, DZ, TW, MD and GT; formal analysis, SD, DZ, JT, TW, RB, DM, YA, RA-T, CC, WS, ME, MD, and GT; funding acquisition, DZ, HM, MD, and GT; investigation, SD, DZ, JT, TW, RB, DM, YA, RA-T, CC, WS, ME, MD, and GT; methodology, DZ, JT, TW, MD, and GT; project administration, DZ, HM, MD, and GT; resources, DZ, HM, MD, and GT; supervision, DZ, MD, and GT; validation, DZ, JT, TW, RB, DM, YA, RA-T, CC,WS, ME,MD, and GT; visualization, SD, DZ, TW, DM, MD, and GT; writing—original draft, SD and GT; writing—review and editing, SD, DZ, TW, DM, HM, MD, and GT. All authors have read and agreed to the published version of the manuscript.

## Funding

This research was funded partially through STTR Phase II contract HDTRA 1-17-C-0030 from the Defense Threat Reduction Agency of the US Department of Defense.

## Acknowledgments

We thank Mr. Michael Woolbert and Dr. Aaron Thompson of the Uniformed Services University of the Health Sciences for their support in performing gamma irradiations.

## Conflict of interest

SJD, RVB, JKT, TJW, DAM, NJPERT, and GJT are employees of Biological Mimetics, Inc. The commercial nature of Biological Mimetics, Inc. does not alter the belief in, or the ability to adhere to the policies of the journal regarding data or material sharing or wider ethical standards.

The remaining authors declare that the research was conducted in the absence of any commercial or financial relationships that could be construed as a potential conflict of interest.

## Publisher’s note

All claims expressed in this article are solely those of the authors and do not necessarily represent those of their affiliated organizations, or those of the publisher, the editors and the reviewers. Any product that may be evaluated in this article, or claim that may be made by its manufacturer, is not guaranteed or endorsed by the publisher.

## Author disclaimer

Material has been reviewed by the Walter Reed Army Institute of Research. There is no objection to its presentation and/or publication. The opinions or assertions contained herein are the private views of the author(s), and are not to be construed as official, or as reflecting true views of the Department of the Army or the Department of Defense. Research was conducted under an IACUC-approved animal use protocol in an AAALAC International - accredited facility with a Public Health Services Animal Welfare Assurance and in compliance with the Animal Welfare Act and other federal statutes and regulations relating to laboratory animals.
